# Digital access and structural indicators of social isolation among older adults in Rwanda: a nationally representative study

**DOI:** 10.3389/fpubh.2026.1867154

**Published:** 2026-09-18

**Authors:** Mignonne Umurerwa, Syed Shafiq Tamal, Yves Gashugi, Fauste Ndikumana

**Affiliations:** 1 Graduate School of Public Health, Yonsei University, Seoul, Republic of Korea; 2 University of Global Health Equity, Butaro, Rwanda; 3 African Center of Excellence in Data Sciences, University of Rwanda, Kigali, Rwanda

**Keywords:** aging, digital access, health equity, living alone, mobile phones, older adults, Rwanda, social isolation

## Abstract

**Background:**

Social isolation and reduced social connectedness are important public health concerns among older adults and are associated with adverse health outcomes. In LMICs, digital expansion may offer new opportunities for social connection; however, evidence from sub-Saharan Africa remains limited.

**Methods:**

We analyzed nationally representative data from the Rwanda Demographic and Health Survey (2019–20), including 2,081 adults aged ≥65 years. Living alone was used as a structural indicator and proxy measure of social isolation. Weighted descriptive statistics and multivariable logistic regression were used to estimate adjusted odds ratios (AORs) with 95% confidence intervals (CIs). Machine learning models (k-nearest neighbors and gradient boosting) were applied as complementary analyses to assess predictive performance and identify influential predictors.

**Results:**

Overall, 9.7% of older adults lived alone and 55.4% owned a mobile phone. Mobile phone ownership was independently associated with lower odds of living alone (AOR = 0.13; 95% CI: 0.08–0.22; *p* < 0.001). Female sex was associated with higher odds of living alone (AOR = 1.86; 95% CI: 1.29–2.67), whereas higher wealth index was associated with lower odds. Machine-learning models demonstrated moderate discriminatory ability (AUC 0.75–0.77) but limited classification performance due to outcome imbalance. Across models, mobile phone ownership consistently emerged as the most influential predictor.

**Conclusion:**

Digital access, operationalized as mobile phone ownership, was strongly associated with reduced likelihood of living alone among older adults in Rwanda. These findings suggest that digital connectivity may contribute to structural dimensions of social integration in rapidly digitizing low- and middle-income settings. Policies that promote equitable access to mobile technologies may support social connectedness and healthy aging among older populations.

## 1 Introduction

Population ageing is accelerating globally, with low- and middle-income countries (LMICs) experiencing the most rapid growth in older adult populations (1). Sub-Saharan Africa, including Rwanda, is undergoing a demographic transition marked by rising life expectancy alongside profound social, economic, and technological change (2). These changes bring increasing attention to social connection, well-being, and healthy ageing, with living alone and reduced social connectedness recognized as important social and health-related concerns in later life (3, 4).

Living alone is a structural characteristic that may reflect one dimension of social isolation but does not capture subjective loneliness, perceived social support, or broader social networks. Social isolation and loneliness are conceptually distinct, and living alone should therefore be interpreted as a structural indicator rather than a comprehensive measure of social isolation (5). Nevertheless, living arrangements are commonly used in population-based research as an objective indicator of household-based social connectedness, particularly when large surveys do not collect validated psychosocial measures (4). Living alone among older adults has also been associated with adverse physical and mental health outcomes, including depression, cognitive decline, functional impairment, and premature mortality (6).

Digital access may be relevant to social connectedness in later life because mobile phones and other information and communication technologies can facilitate communication with family members, intergenerational contact, and participation in social and community networks (7, 8). However, digital access encompasses more than ownership; digital skills, meaningful use, perceived utility, and affordability may also shape the benefits derived from technology (9, 10). Household mobile phone ownership nevertheless represents an important and measurable indicator of basic digital access in settings where access to smartphones, internet services, and advanced digital literacy remains uneven.

Evidence on digital access and social connectedness among older adults in sub-Saharan Africa remains limited. Older adults in the region may experience overlapping socioeconomic and geographic vulnerabilities, including poverty, lower educational attainment, rural residence, and limited access to formal social protection systems (11). Studies from Ghana, South Africa, and Kenya suggest that mobile phones may be relevant to social connectedness and participation among older adults (12). However, nationally representative evidence examining the association between household mobile phone ownership and living alone among older adults remains scarce.

Rwanda provides an important context for examining this association. The country has undergone substantial digital transformation, with expanded mobile connectivity and national efforts to promote digital inclusion (13). The 2019–20 Rwanda Demographic and Health Survey (RDHS) reported that approximately 78% of households owned at least one mobile phone, although access varies across socioeconomic and demographic groups (14, 15). At the same time, demographic change, migration, and changing household structures may be influencing traditional patterns of family support for older adults (16). Understanding how household digital access relates to living arrangements may therefore contribute to discussions of healthy ageing, social protection, and equitable digital transformation.

This study examined the association between household mobile phone ownership and living alone as a structural indicator and proxy measure of social isolation among adults aged 65 years and older in Rwanda, using nationally representative data from the 2019–20 RDHS. Because the RDHS does not collect validated measures of loneliness, perceived social support, or broader social network characteristics, living alone was operationalized as a structural indicator of social isolation. The study primarily used multivariable logistic regression to estimate adjusted associations, with complementary exploratory machine-learning analyses used to examine predictive performance and patterns among the selected predictors.

## 2 Methods

### 2.1 Data source

This study used data from the 2019–20 Rwanda Demographic and Health Survey (RDHS), a nationally representative demographic and household survey conducted by the National Institute of Statistics of Rwanda (NISR) in collaboration with the Ministry of Health and ICF International.

The RDHS employs a stratified two-stage cluster sampling design. In the first stage, enumeration areas are selected with probability proportional to size, followed by systematic sampling of households within clusters. All analyses incorporated DHS sampling weights, clustering, and stratification to produce nationally representative estimates and correct standard errors.

The analysis drew on the Person Recode (PR) dataset, which contains individual-level information for all usual household members listed in sampled households. The PR dataset provided individual-level information on household membership, living arrangements, demographic characteristics, socioeconomic characteristics, and household mobile phone ownership, allowing these factors to be examined among older adults.

### 2.2 Study population

The analytic sample comprised adults aged 65 years and older residing in Rwanda. Although age 60 years and older is also commonly used to define older adults, a threshold of ≥65 years has been used in demographic research on older populations in sub-Saharan Africa (17). This study therefore focused on adults aged ≥65 years to examine a more advanced older-age population. Individuals were eligible if they were aged ≥65 years and were listed as usual household members in the sampled households. A total of 2,081 individuals identified in the 2019–20 Rwanda Demographic and Health Survey (RDHS) Person Recode (PR) dataset met the age criterion and were included in the analysis. No additional eligibility exclusions were applied, and no observations were excluded because of missing data in the variables included in the analysis.

The public-use RDHS datasets accessed for this study contained complete information for all variables included in the analysis. Therefore, no observations were excluded because of missing data. DHS sampling weights were applied to all descriptive and inferential analyses to ensure national representativeness of the estimates.

The RDHS implements rigorous field supervision, standardized data collection procedures, and multiple quality-control checks during data processing, contributing to the completeness and reliability of the public-use datasets.

### 2.3 Measures and variables

#### 2.3.1 Outcome variable: living alone as a structural indicator of social isolation

The outcome variable was living alone, derived from the RDHS household size variable (hv009). Participants living in one-person households (hv009 = 1) were classified as living alone, whereas those living in households with two or more members (hv009 ≥ 2) were classified as not living alone. Household size was used solely to derive the outcome variable and was not included as an independent explanatory variable in the regression or machine-learning analyses. This classification reflects the respondent’s household composition at the time of the survey, as recorded in the DHS household roster.

Living alone represents a structural indicator and proxy measure of social isolation. Although it does not fully capture subjective experiences such as loneliness, perceived social support, or broader social network engagement, it is widely used in population-based and demographic research when validated psychosocial measures are unavailable. Previous research has also demonstrated that living alone is associated with adverse health outcomes and serves as an important indicator of social vulnerability among older adults. Accordingly, living alone provides a practical structural measure for population-based analyses, particularly in large national surveys such as the Demographic and Health Surveys (DHS) (18, 19, 20).

This approach is consistent with population-based research in low- and middle-income settings where direct measures of loneliness, social participation, or social network characteristics are not routinely collected (21). The classification reflects the respondent’s household composition at the time of the survey; because it is based solely on household size, it does not capture social relationships outside the household or the quality and frequency of social interactions, and may not fully reflect atypical living arrangements.

#### 2.3.2 Primary exposure: household Mobile phone ownership

The primary exposure variable was household mobile phone ownership, derived from the RDHS household variable hv243a, which was merged into the Person Recode (PR) dataset using the standard RDHS household identifiers. The variable indicates whether the respondent resided in a household that owned at least one mobile phone at the time of the survey. Because household mobile phone ownership reflects the digital environment in which older adults live, it was used as the primary indicator of digital access in this study. Respondents were categorized as follows:

- 1 = respondent resides in a household that owns at least one mobile phone
- 0 = respondent resides in a household that does not own a mobile phone

Household mobile phone ownership was selected because it represents the most basic and widely available form of digital access available in the RDHS 2019–20 dataset.

#### 2.3.3 Covariates

Based on prior literature and theoretical relevance, the pre-specified covariates included age, sex, wealth index, education level, and place of residence. These variables were selected because they represent key demographic, socioeconomic, and geographic characteristics that may be associated with both household mobile phone ownership and living arrangements among older adults. Age was modeled as a continuous variable (measured in completed years) in the regression and machine-learning analyses to preserve statistical information and power. Age was categorized into four groups (65–69, 70–74, 75–79, and ≥80 years) in the regression and machine-learning analyses, with 65–69 years as the reference category, and the same categories were used for descriptive presentation in Table 1. The remaining covariates were included as categorical variables to adjust for potential confounding.

**Table 1:** Sample characteristics of adults aged ≥65 years, Rwanda RDHS 2019–20.

Characteristic	Frequency (*N*)	Percentage (%)
Age (years)		
Median (IQR)	71 (65, 98)	
Mean (SD)	73.55 (7.5)	
65–69	780	37.5
70–74	525	25.2
75–79	402	19.3
≥80	374	18.0
Sex		
Male	827	39.7
Female	1,254	60.3
Wealth index		
Poorest	523	25.1
Poorer	517	24.8
Middle	401	19.3
Richer	364	17.5
Richest	276	13.3
Residence		
Rural	1790	86.0
Urban	291	14.0
Education level		
No education	1,167	56.1
Primary education	815	39.2
Secondary education	87	4.1
Higher education	12	0.6
Phone ownership		
Owns a mobile phone	1,152	55.4
No mobile phone	929	44.6
Living arrangement		
Lives with others	1880	90.3
Lives alone	201	9.7
Total	2081	100

Values are presented as frequencies (N) and weighted percentages (%). Percentages are calculated using DHS sampling weights and may not sum to 100 due to rounding. SD, standard deviation; IQR, interquartile range.

Household composition was not included as a separate covariate because the primary outcome, living alone, was directly constructed from household size (hv009 = 1); therefore, adjusting for household composition would overlap directly with the outcome definition. Occupational variables available in the RDHS dataset were reviewed but were not included in the final model because of substantial heterogeneity and missingness among adults aged 65 years and older. Marital status and region were also available in the RDHS dataset but were not included in the final pre-specified covariate set.

### 2.4 Statistical analysis

All statistical and machine-learning analyses were conducted using R version 4.4.1 (R Foundation for Statistical Computing, Vienna, Austria). The survey-weighted analyses were conducted using the survey package (version 4.5), while machine-learning analyses and model evaluation used caret (version 7.0.1), xgboost (version 3.2.1.1), and pROC (version 1.19.0.1). Data management and transformation were performed using dplyr (version 1.2.1).

Weighted descriptive statistics were used to summarize participant characteristics and estimate the prevalence of living alone and mobile phone ownership among adults aged ≥65 years. The outcome variable, living alone, was defined using the RDHS household size variable (hv009 = 1), with participants living in households with two or more members classified as not living alone. Household mobile phone ownership was derived from the RDHS variable hv243a. Covariates included age, sex, educational attainment, household wealth index, and place of residence. Educational attainment was categorized as no education, primary, secondary, and higher education.

Multivariable logistic regression was used to estimate adjusted associations between household mobile phone ownership and living alone while controlling for potential confounders. The binary outcome was coded as 1 for participants living alone (household size = 1; hv009 = 1) and 0 for participants living with two or more household members. Household mobile phone ownership (hv243a) was coded as 1 for households owning a mobile phone and 0 for households without a mobile phone. Age was entered as a continuous variable in completed years, while sex, wealth index, education level, and place of residence were entered as categorical variables. The reference categories were male for sex, poorest for wealth index, no education for education level, and rural for place of residence. DHS sampling weights (hv005) were applied to the analyses and normalized by dividing by 1,000,000 in accordance with DHS analytic recommendations. The complex survey design was accounted for using the clustering and stratification variables provided in the RDHS dataset. Adjusted odds ratios (AORs), 95% confidence intervals (CIs), and *p*-values were reported.

No missing values were identified for the variables included in the final analytic dataset; therefore, no participants were excluded because of missing data.

Model diagnostics were conducted to assess multicollinearity and model calibration. Variance inflation factors (VIFs) were examined to assess potential multicollinearity among predictors. Because the primary regression model accounted for the complex RDHS sampling design, model fit was evaluated using calibration plots rather than the standard Hosmer–Lemeshow test, which is not directly designed for survey-weighted regression models. The multivariable regression was fitted using the survey package in R (version 4.5) with the svydesign()function to specify sampling weights, primary sampling units, and strata, followed by the svyglm() function with a quasibinomial family. Regression results were interpreted in terms of the direction, magnitude, and statistical significance of associations.

### 2.5 Machine learning models

To complement the regression-based analysis, two machine-learning approaches, k-nearest neighbors (KNN) and gradient boosting (XGBoost), were included as exploratory analyses. The primary purpose of these models was not to establish causal relationships or replace regression-based inference, but to assess predictive discrimination and explore whether nonlinear patterns and differences in the relative contribution of predictors could provide additional insight into classification of living-alone status. The same outcome definition and predictor variables were used across the regression and machine-learning analyses to facilitate comparison of results. Unlike the descriptive and regression analyses, the machine-learning models did not incorporate RDHS sampling weights, primary sampling units, or sampling strata; therefore, their results were interpreted as exploratory predictive analyses within the analytic sample rather than as nationally representative predictive estimates.

Predictor variables included household mobile phone ownership, age, sex, wealth index, education level, and place of residence. The dataset was divided into training (70%) and testing (30%) subsets using stratified sampling to preserve the proportion of participants living alone in both subsets. The training set included 1,457 participants, of whom 141 (9.68%) were living alone, while the testing set included 624 participants, of whom 60 (9.62%) were living alone.

For KNN, predictors were represented numerically, with categorical variables converted to indicator variables. Age was centered and scaled using parameters estimated from the training data and then applied unchanged to the testing data; categorical predictors were represented using indicator variables. Candidate neighborhood sizes (k = 1, 3, 5, 7, 9, 11, 15, 21, and 31) were evaluated using the area under the receiver operating characteristic curve (AUC) on the testing subset. The value k = 31 yielded the highest observed test-set AUC and was therefore selected for the final exploratory KNN analysis. Because the testing subset was also used for selection of the neighborhood size, the resulting performance estimates were considered exploratory and may be subject to optimistic estimation of out-of-sample performance.

For the gradient boosting model, an Extreme Gradient Boosting (XGBoost) classifier with a binary logistic objective function was trained using the training dataset. Class imbalance was addressed by incorporating the scale_pos_weight parameter, calculated from the ratio of non-isolated to isolated participants in the training dataset. The model was specified with a learning rate (eta) of 0.05, maximum tree depth of 4, minimum child weight of 1, subsampling fraction of 0.8, and column subsampling fraction of 0.8, with a maximum of 400 boosting rounds and early stopping after 30 rounds without improvement in test-set AUC. The testing dataset was therefore used both for monitoring model performance during training and for subsequent performance evaluation. Model performance was assessed using the area under the receiver operating characteristic curve (AUC). Feature importance was examined using model gain to assess the relative contribution of each predictor to the classification of living alone as a structural indicator of social isolation.

Machine-learning findings were interpreted cautiously as exploratory predictive analyses, designed to provide additional insight into classification performance and predictor importance rather than to replace or confirm the regression-based inferential findings.

### 2.6 Ethical considerations and data availability

This study relied exclusively on publicly available, anonymized data from the Demographic and Health Surveys (DHS) Program and was exempt from institutional review board approval. Access to the Rwanda DHS data was granted by the DHS Program on October 24, 2025, for this registered research project.

The DHS public-use datasets contain no personally identifiable information. Names, household addresses, and precise geographic identifiers are removed, and any geographic coordinates collected are displaced to prevent identification of enumeration areas. As such, the analysis posed no risk to respondents, and no attempt was made to identify any individual, household, or community.

In accordance with DHS data-use agreements, the data were used solely for statistical reporting and analysis related to the registered research purpose. Redistribution of micro-level RDHS data was not undertaken. Given the use of anonymized secondary data, this study qualifies for ethical review exemption, consistent with institutional and journal requirements.

## 3 Results

### 3.1 General characteristics

A total of 2,081 Rwandan adults aged 65 years and older were included in the analytic sample. Unless otherwise specified, frequencies (N) are presented as unweighted counts, whereas percentages and prevalence estimates are weighted using the RDHS sampling weights to ensure national representativeness.

The weighted mean age was 73.55 years (IQR: 65, 98), and women comprised 60.3% of the weighted study population.

Overall, the weighted prevalence of living alone was 9.7%, while 55.4% of older adults reported household mobile phone ownership. Table 1 presents the characteristics of the study population.

### 3.2 Descriptive characteristics of the study population

The socio-demographic characteristics of adults aged 65 years and older are summarized in Table 1. Women constituted a larger proportion of the study population (60.3%) compared with men (39.7%). The age distribution was concentrated in the younger-old groups, with 37.5% aged 65–69 years and 25.2% aged 70–74 years, while progressively smaller proportions were observed in older age categories.

Most participants resided in rural areas (86.0%), reflecting the predominantly rural population structure of Rwanda. In terms of socioeconomic status, a large proportion of older adults were concentrated in the lower wealth quintiles, with 25.1% in the poorest quintile and 24.8% in the poorer quintile.

Mobile phone ownership was reported by 55.4% of older adults, while 44.6% did not own a mobile phone, indicating a notable digital access divide within this population. Regarding living arrangements, 9.7% of participants lived alone, while the majority (90.3%) lived with other household members.

Educational attainment was generally low, with more than half of the participants reporting no formal education, reflecting historical disparities in access to schooling among older generations in Rwanda.

### 3.3 Bivariate association between mobile phone ownership and living alone

Bivariate analysis using Pearson’s chi-square test demonstrated a statistically significant association between household mobile phone ownership and living alone among adults aged 65 years and older (Pearson’s χ^2^ = 179.6, df = 1, *p* < 0.001).

As shown in Table 2, living alone was substantially more common among older adults residing in households without a mobile phone than among those living in households with at least one mobile phone. Specifically, 19.4% of individuals residing in households without a mobile phone lived alone, compared with 1.8% among those residing in households with a mobile phone. Conversely, 98.2% of individuals residing in households with a mobile phone lived with others, compared with 80.6% among those residing in households without a mobile phone.

**Table 2:** Living arrangements (lives alone vs. lives with others) by household mobile phone ownership among older adults (≥65 years), RDHS 2019–20.

Household phone ownership	Lives with others	Lives alone
No phone	80.6%	19.4%
Owns phone	98.2%	1.8%

Pearson’s χ^2^ = 179.6, df = 1, *p* < 0.001.

The distribution of living arrangements by mobile phone ownership is presented in Figure 1. The figure illustrates the percentage distribution of living arrangements stratified by mobile phone ownership status. Living alone was used as a structural indicator and proxy measure of social isolation.

**Figure 1:**
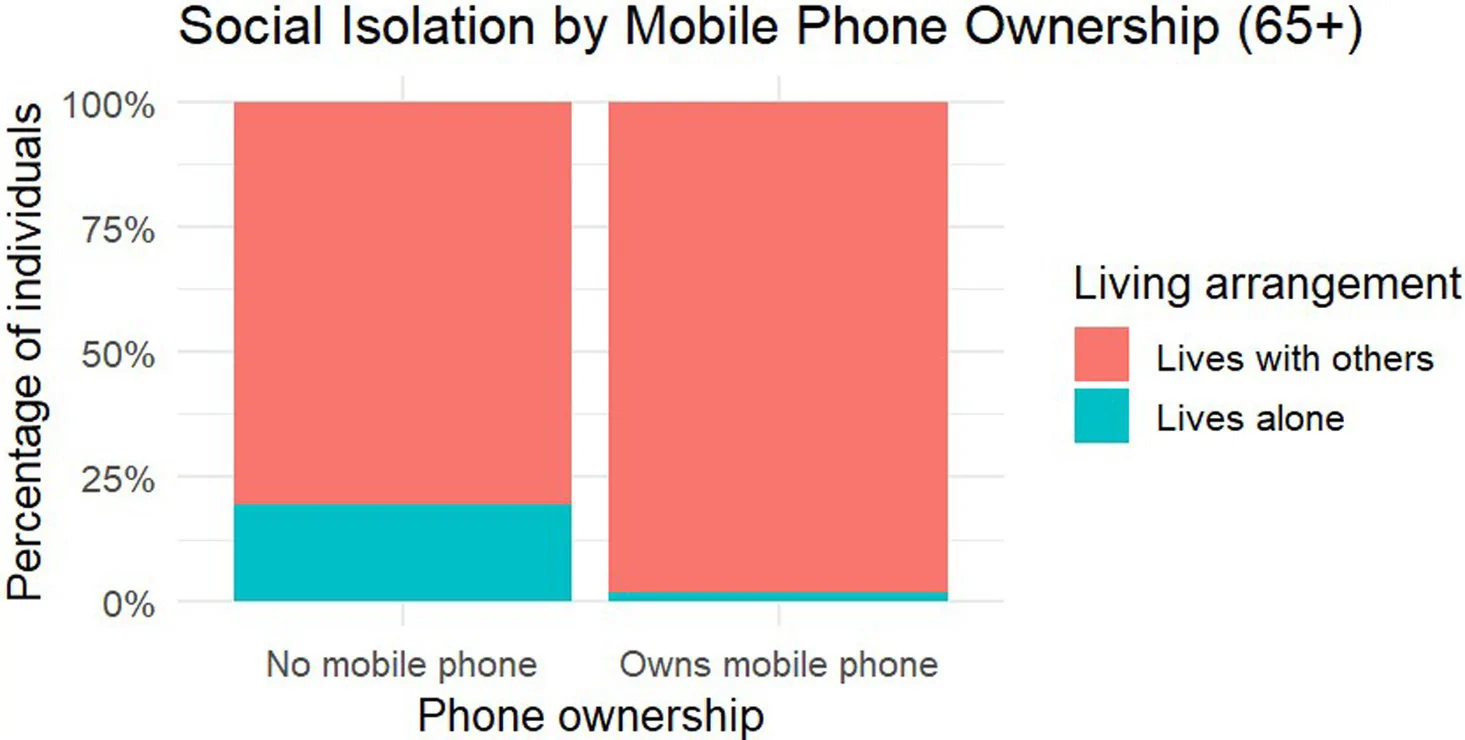
Living arrangement status by household mobile phone ownership among older adults aged ≥65 years, Rwanda DHS 2019–20.

### 3.4 Unadjusted logistic regression analysis

In unadjusted logistic regression analysis, mobile phone ownership was strongly associated with living alone among older adults. Individuals aged 65 years and older who owned a mobile phone had substantially lower odds of living alone compared with those who did not own a phone (OR = 0.08, 95% CI: 0.05–0.12, *p* < 0.001). This corresponds to an approximate 92% reduction in the odds of living alone among mobile phone owners.

This pronounced crude association indicates a strong relationship between mobile phone ownership and living arrangements among older adults. However, because mobile phone ownership may be correlated with other socio-demographic characteristics, multivariable logistic regression models were subsequently estimated to assess whether this association persisted after adjustment for age, sex, socioeconomic status, education level, and place of residence.

### 3.5 Multivariable logistic regression results

Results from the multivariable logistic regression model presented in Table 3, showed that mobile phone ownership was strongly and inversely associated with living alone. Older adults who lived in households that owned a mobile phone had 87% lower odds of living alone compared with those living in households without a mobile phone (AOR = 0.13; 95% CI: 0.08–0.22; *p* < 0.001).

**Table 3:** Adjusted odds of living alone among older adults (≥65 years).

Variables	Adjusted odds ratio (AOR)	95% Confidence interval		*p*-value
Mobile phone ownership				
Does not own a mobile phone	1.00 (ref)	0.08	0.22	<0.001
Owns a mobile phone	0.12			
Age (years)				
65–69	1.00 (ref)			
70–74	1.18	0.82	1.69	0.370
75–79	1.31	0.88	1.95	0.180
≥80	1.47	0.99	2.18	0.056
Sex				
Male	1.00 (ref)	1.29	2.67	0.001
Female	1.86			
Wealth index				
Poorest	1.00 (ref)			
Poorer	0.88	0.61	1.27	0.497
Middle	0.72	0.48	1.09	0.120
Richer	0.56	0.35	0.88	0.013
Richest	0.43	0.24	0.77	0.004
Education level				
No education	1.00 (ref)			
Primary education	0.78	0.40	1.41	0.433
Secondary education	0.77	0.43	1.30	0.347
Higher education	1.26	0.74	2.08	0.377
Place of residence				
Rural	1.00 (ref)	0.70	2.58	0.376
Urban	1.34			

Adjusted odds ratios (AORs) were estimated using multivariable logistic regression models accounting for the complex survey design and sampling weights of the Rwanda Demographic and Health Survey (2019–20). The model was adjusted for age, sex, wealth index, education level, and place of residence. Reference (ref) categories are indicated. CI, confidence interval.

Sex and socioeconomic status were also significant predictors. Older women had significantly higher odds of living alone compared with men (AOR = 1.86; 95% CI: 1.30–2.69; *p* < 0.001). Higher wealth index was associated with lower likelihood of living alone, with each increase in wealth quintile associated with a 26% reduction in the odds of living alone (AOR = 0.74; 95% CI: 0.62–0.89; *p* = 0.001).

Compared with adults aged 65–69 years, the adjusted odds of living alone increased across older age groups but did not reach statistical significance. Adults aged ≥80 years had the highest odds of living alone (AOR = 1.47; 95% CI: 0.99–2.18; *p* = 0.056), although this association was of borderline statistical significance. Neither education level nor place of residence was significantly associated with living alone in the adjusted model.

The adjusted odds ratios for factors associated with living alone are presented in Figure 2. A forest plot of adjusted odds ratios (AORs) and 95% confidence intervals from a weighted multivariable logistic regression model is shown. The model adjusts for age, sex, wealth index, education level, and place of residence. The dashed vertical line indicates an odds ratio of 1.0.

**Figure 2:**
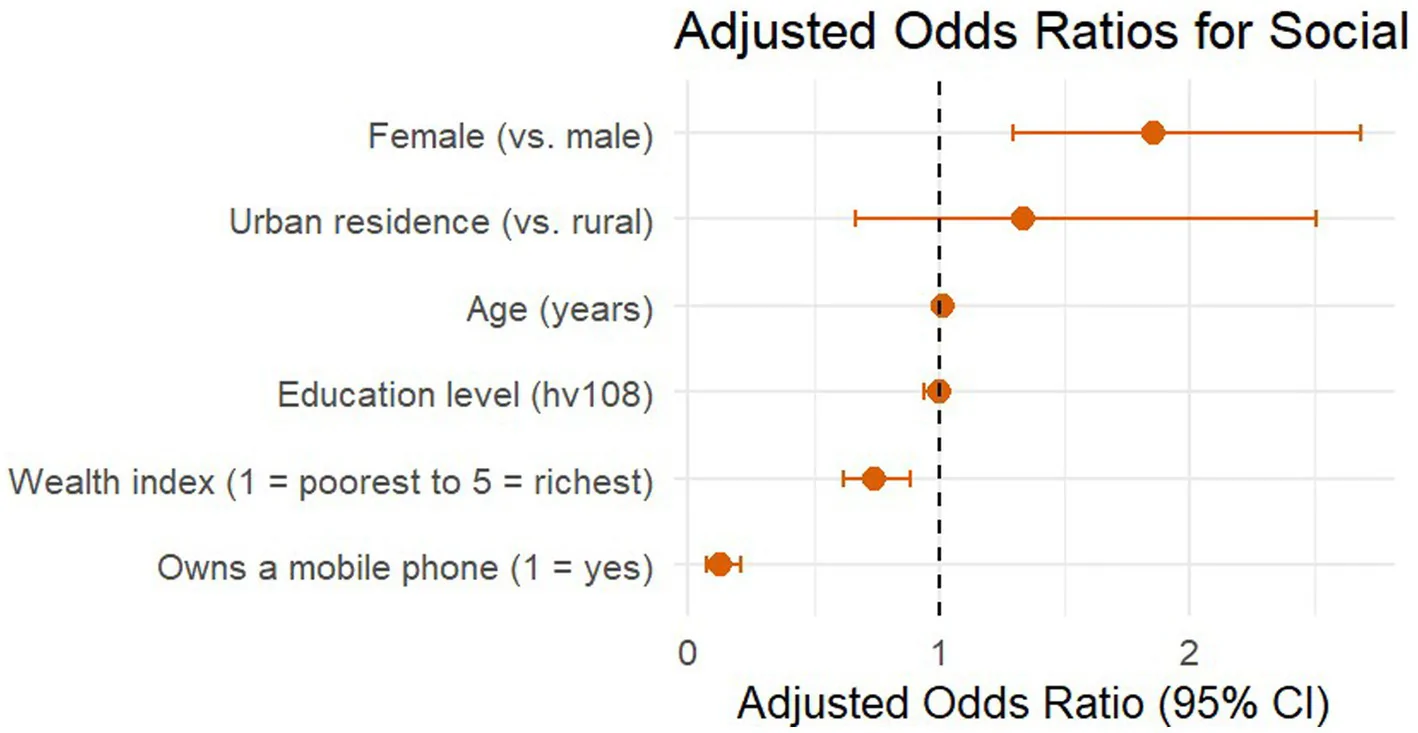
Adjusted odds ratios for living alone among older adults aged ≥65 years, Rwanda DHS 2019–20.

Model-based predicted probabilities of living alone stratified by mobile phone ownership are shown in Figure 3. Predicted probabilities were derived from a multivariable logistic regression model adjusting for age, sex, wealth index, education level, and place of residence.

**Figure 3:**
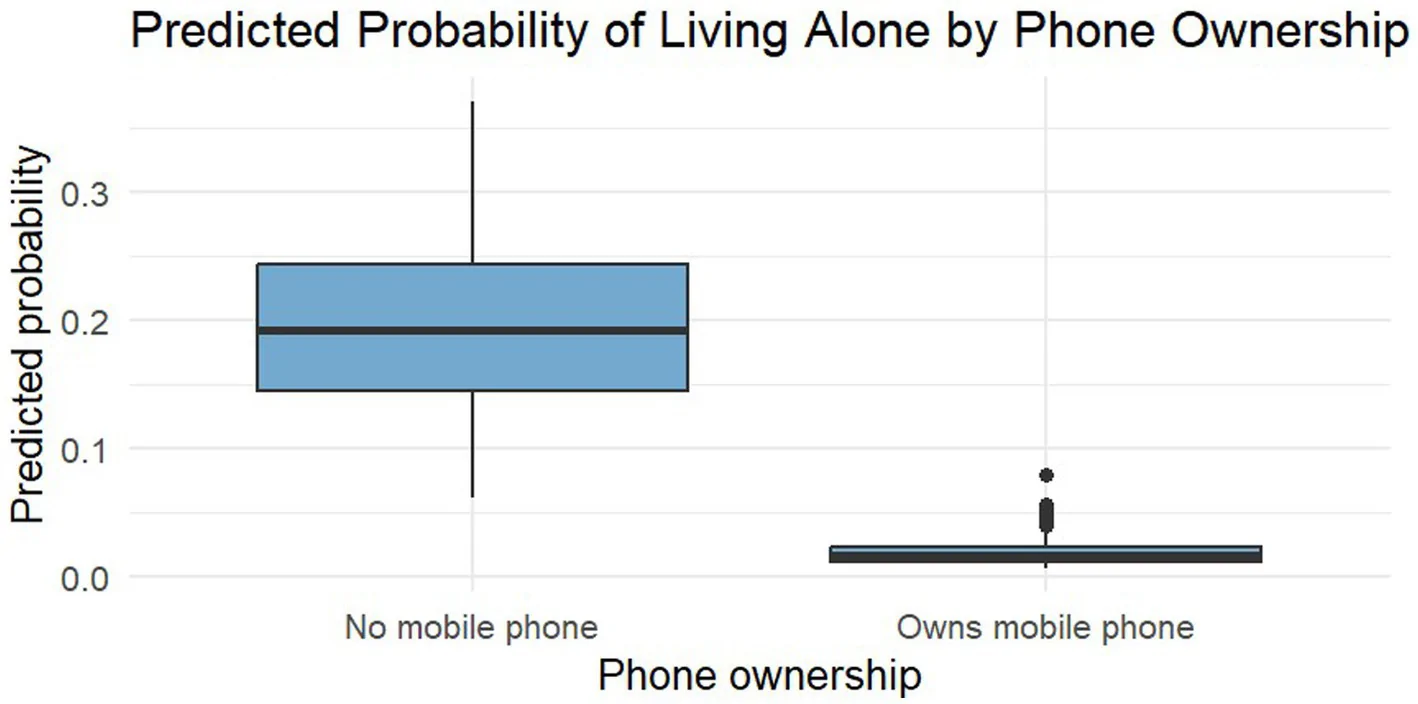
Predicted probability of living alone by mobile phone ownership among older adults aged ≥65 years, Rwanda DHS 2019–20.

### 3.6 Machine learning results

To complement the regression-based findings, machine-learning models were used to evaluate the predictive performance of sociodemographic and digital-access variables in distinguishing older adults who lived alone from those who lived with others. Results from the k-nearest neighbors (KNN) and gradient boosting (XGBoost) models are presented below.

#### 3.6.1 K-nearest neighbors (KNN)

To explore potential non-linear patterns associated with living arrangements among older adults, a supervised machine learning approach using the K-Nearest Neighbors (KNN) algorithm was applied. The model was trained on DHS data in which approximately 9.6% of participants lived alone. The dataset was split into training (70%) and testing (30%) subsets using stratified sampling to preserve the prevalence of living alone across datasets. The outcome distribution remained highly imbalanced, with individuals living alone comprised approximately one-tenth of the sample in both subsets.

Given the substantial class imbalance, no resampling was performed; instead, model performance was evaluated using the area under the receiver operating characteristic curve (AUC), a metric that is robust to imbalanced outcome distributions. This approach ensured that model discrimination was assessed independently of classification thresholds while maintaining the original population structure.

#### 3.6.2 Model performance

Model performance was evaluated across candidate neighborhood sizes (k = 1, 3, 5, 7, 9, 11, 15, 21, and 31) using the area under the receiver operating characteristic curve (AUC) calculated on the testing subset. The highest observed AUC was obtained at k = 31 (AUC = 0.76), which was selected for the final exploratory KNN analysis. Because the testing subset was used for selection of k, the reported AUC should be interpreted as an exploratory performance estimate rather than a definitive estimate of independent out-of-sample performance. The tuning of the KNN model, based on test-set AUC across the candidate values of k, is presented in Figure 4. Predictive performance generally improved as k increased and plateaued at higher neighborhood sizes. Smaller k values may be more sensitive to local variation, whereas larger k values produce smoother decision boundaries. The plateauing of AUC beyond approximately k = 15 suggests diminishing gains from further increases in neighborhood size.

**Figure 4:**
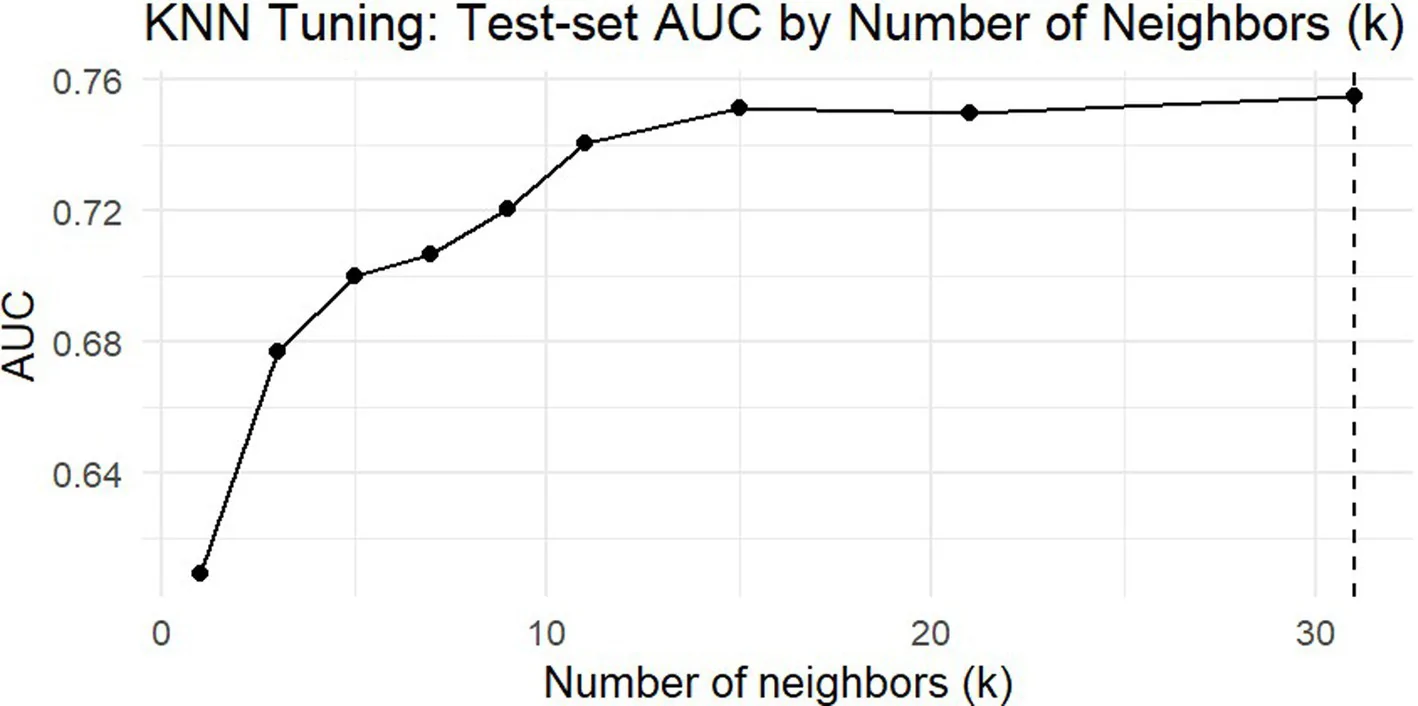
KNN tuning curve showing test-set AUC by number of neighbors (*k*).

#### 3.6.3 Classification performance of the final KNN model

Using the optimal neighborhood size (k = 31), the final KNN model achieved an overall classification accuracy of 90.4% on the test dataset. Specificity for identifying individuals who lived with others was 100%, while sensitivity for identifying individuals who lived alone was 0%, resulting in a balanced accuracy of 0.50. The high overall accuracy reflects the predominance of individuals who lived with others rather than effective identification of those who lived alone.

The performance metrics of the K-nearest neighbors (KNN) model are summarized in Table 4. The model demonstrated high specificity but very low sensitivity, indicating limited ability to correctly identify individuals who lived alone.

**Table 4:** Performance of the K-nearest neighbors model.

Metric	Value
Optimal number of neighbors (k)	31
Area under ROC curve (AUC)	0.76
Accuracy	0.90
Sensitivity	0.00
Specificity	1.00
Balanced accuracy	0.50
Prevalence of living alone	0.096

Living alone was used as a structural indicator and proxy measure of social isolation.

70/30 stratified split → training-based standardization → candidate k values evaluated on test set → k = 31 selected → final test performance reported → explicitly acknowledge that test-set tuning can make performance optimistic.

#### 3.6.4 Receiver operating characteristic (ROC) analysis

The ROC curve for the final KNN model is shown in Figure 5. The curve lies consistently above the diagonal reference line, yielding an AUC of 0.755 and indicating some ability to discriminate between participants living alone and those not living alone across classification thresholds, despite the model’s failure to identify living-alone cases at the reported classification threshold. This suggests that the combination of mobile phone ownership, age, sex, wealth, education, and residence contains meaningful information for distinguishing individuals who live alone from those who live with others.

**Figure 5:**
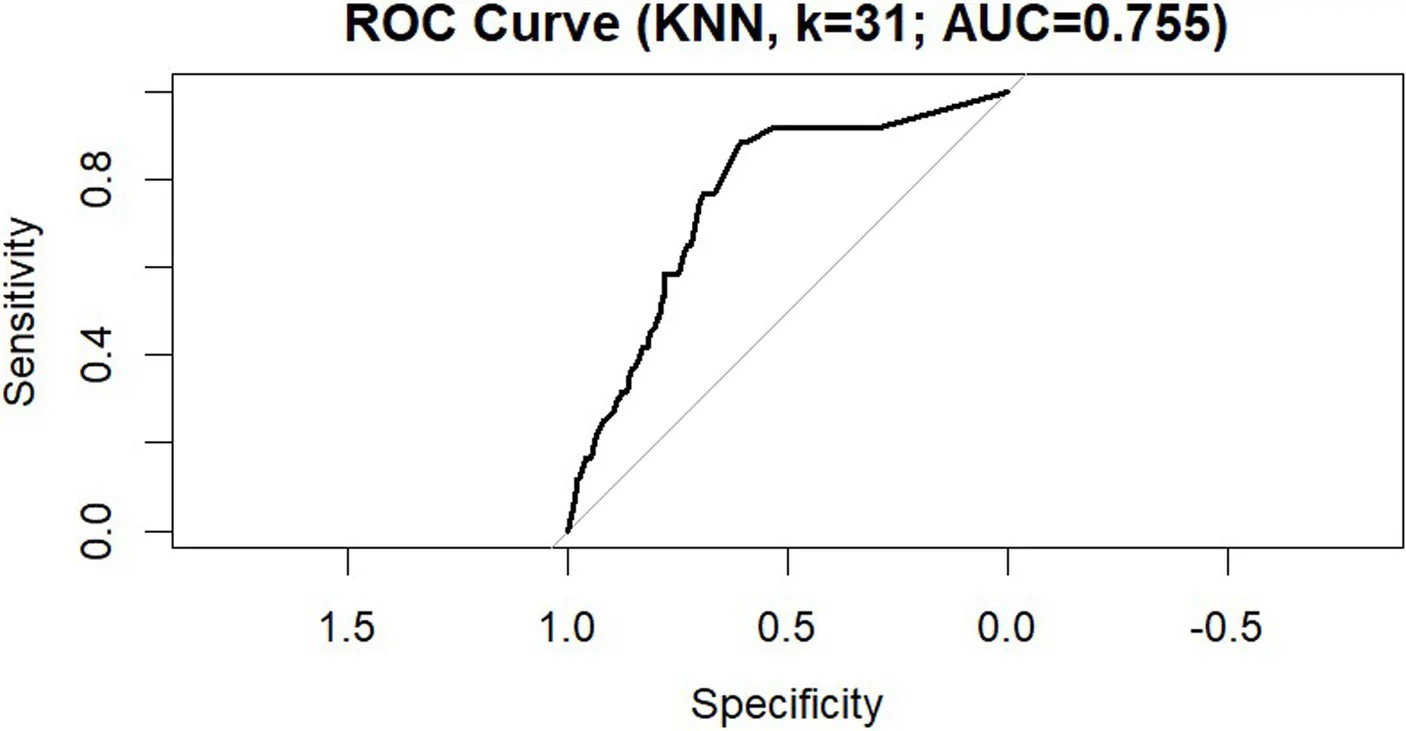
Receiver operating characteristic (ROC) curve for the final KNN model (*k* = 31).

#### 3.6.5 Confusion matrix and error structure

The classification performance of the KNN model is summarized in Figure 6. The confusion matrix shows that the model correctly classified all individuals who lived with others in the test set but failed to identify any individuals who lived alone, misidentifying all isolated cases as non-isolated. As a result, the model achieved high overall accuracy (90.4%) and perfect specificity (100%), but zero sensitivity for detecting participants who lived alone.

**Figure 6:**
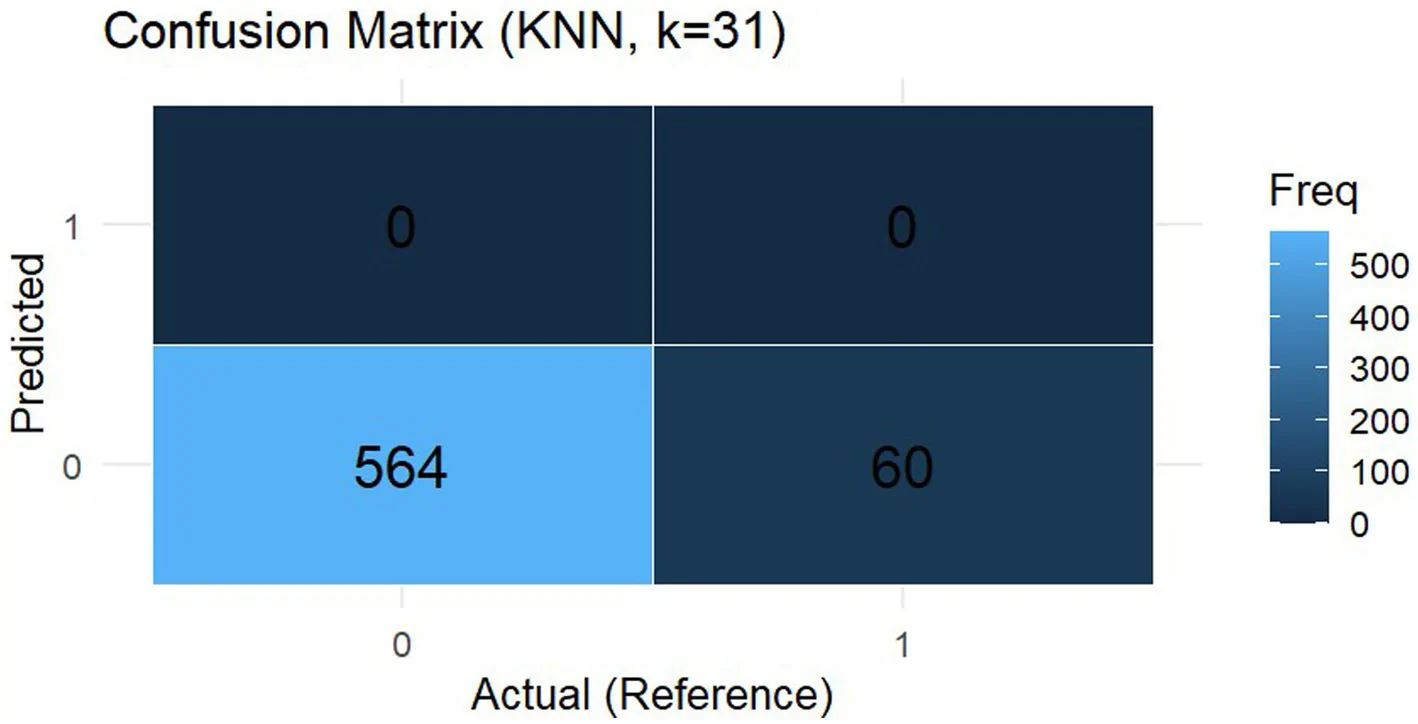
Confusion matrix for the KNN model (*k* = 31).

This pattern reflects the substantial class imbalance in the dataset, where approximately 9.6% of older adults lived alone. In such settings, distance-based algorithms like KNN tend to favor the majority class, particularly when larger k values are selected. Consequently, despite acceptable AUC performance, the KNN model was not effective for identifying individuals at highest risk of isolation.

#### 3.6.6 Structure of the predictor space

The distribution of standardized predictors by living-alone status is visualized using principal component analysis in Figure 7. A principal component analysis (PCA) of the standardized predictors showed substantial overlap between individuals who lived alone and those who lived with others in the reduced two-dimensional feature space, with no clear clustering by living-alone status.

**Figure 7:**
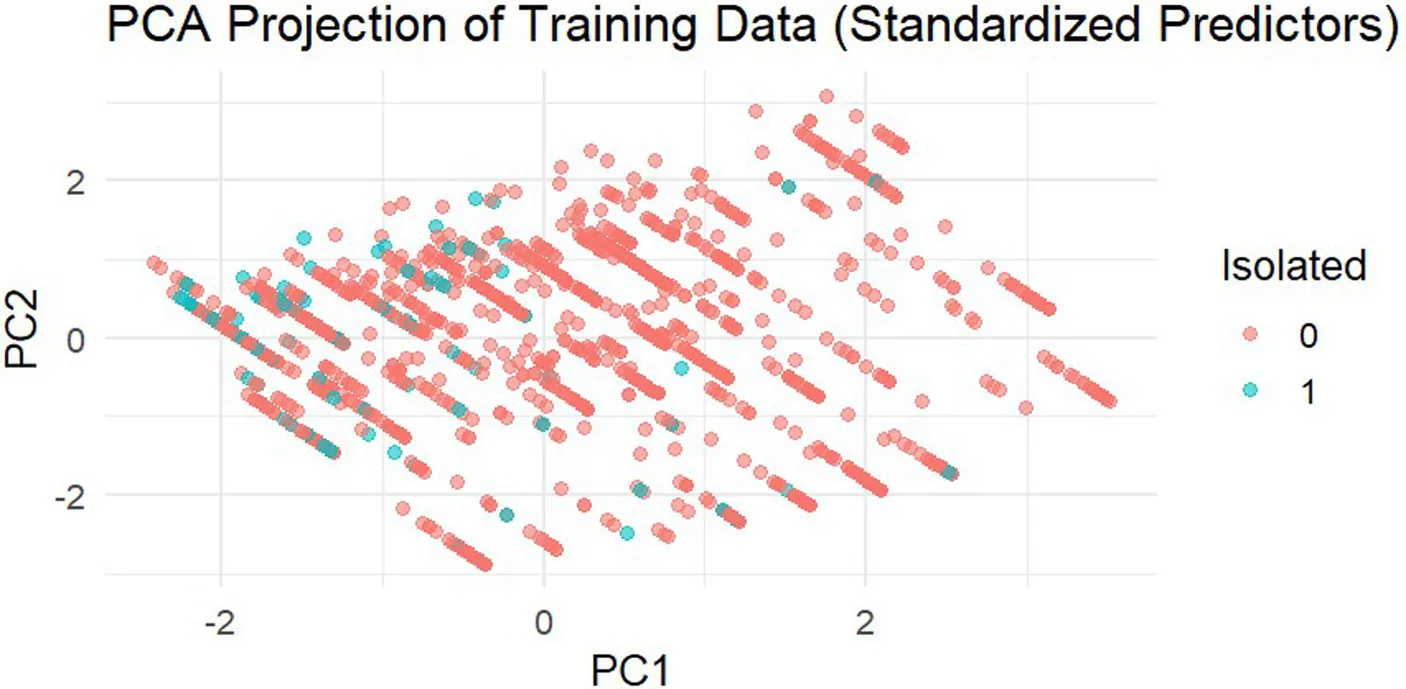
PCA projection of standardized predictors by living-alone status among older adults aged ≥65 years, Rwanda DHS 2019–20.

This lack of visual separability indicates that living-alone status is not strongly separated in the reduced feature space defined by the available predictors. The overlap helps explain the KNN model’s poor sensitivity, as distance-based classification relies on clear neighborhood separation between outcome classes. These findings suggest that structural dimensions of social isolation among older adults are influenced by complex and overlapping social determinants rather than easily separable patterns in observed covariates.

#### 3.6.7 Gradient boosting (XGBoost)

To explore non-linear relationships and interactions among predictors of living alone, a gradient boosting model (XGBoost) was used. The model was trained on 70% of the analytic sample and evaluated on a held-out 30% test set. Given the relatively low prevalence of living alone (9.7%), class imbalance was addressed using a scale-positive-weight parameter.

Model performance was assessed using the area under the receiver operating characteristic curve (AUC), and feature importance metrics were examined to identify key predictors. The performance of the XGBoost model is illustrated in Figure 8. On the test set, the model demonstrated good discriminatory ability, with an AUC of 0.77, indicating improved classification compared to chance.

**Figure 8:**
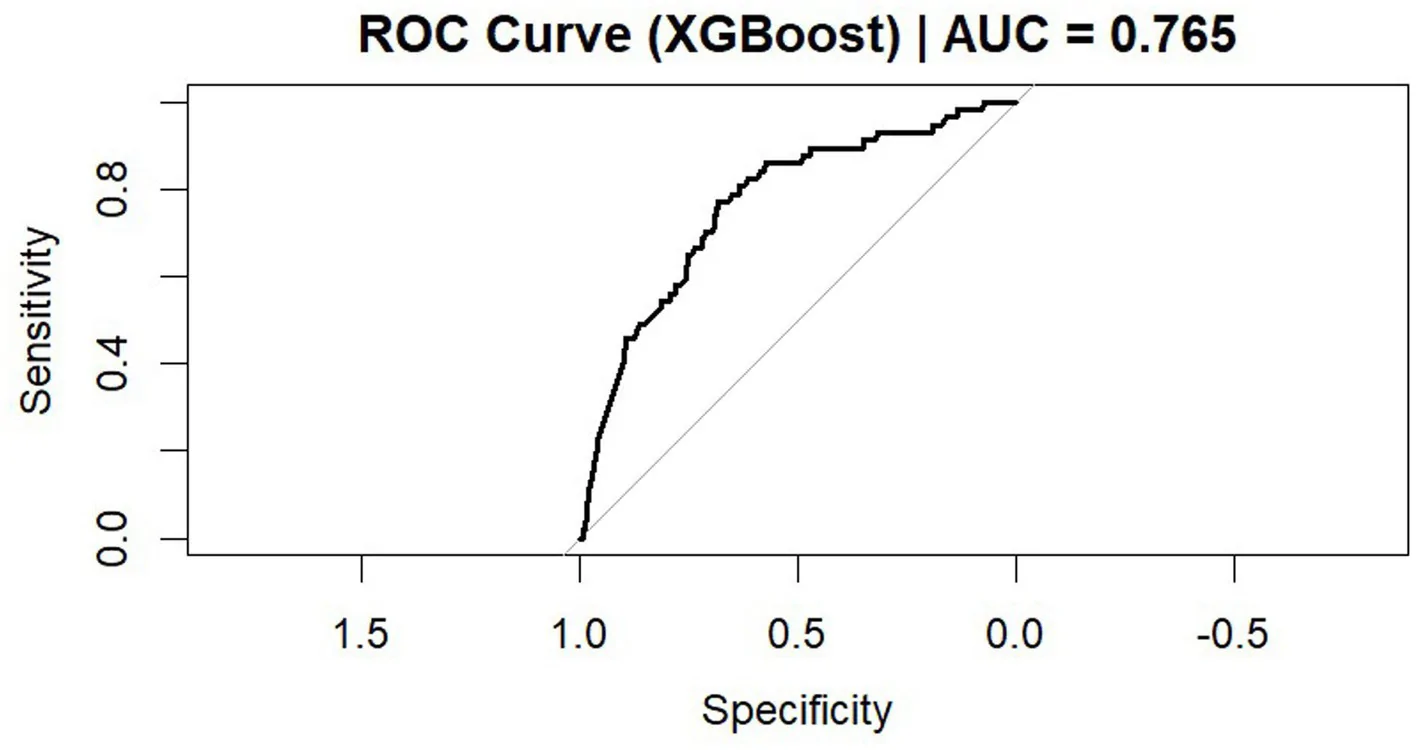
Receiver operating characteristic (ROC) curve for the XGBoost model.

At a classification threshold of 0.30, selected to improve detection of the relatively rare outcome, the model achieved high sensitivity (1.00) but low specificity (0.00), resulting in a balanced accuracy of 0.50. This reflects a tendency to classify most individuals as at risk at lower thresholds, a common trade-off in imbalanced datasets. Overall accuracy was low (9.1%) due to a high number of false positives; therefore, AUC was considered the primary performance metric.

#### 3.6.8 Feature importance

The relative importance of predictors in the XGBoost model is presented in Figure 9. According to the gain-based feature importance metric, mobile phone ownership was the highest-ranked predictor, contributing approximately 49% of the total model gain and exceeding all other predictors. Wealth index was the second most influential predictor, followed by age, whereas sex, education level, and place of residence contributed relatively less to model’s predictive performance. These feature-importance measures reflect the relative contribution of predictors to the fitted XGBoost model’s predictive performance and should not be interpreted as evidence of causal importance, independent effects, or statistical significance.

**Figure 9:**
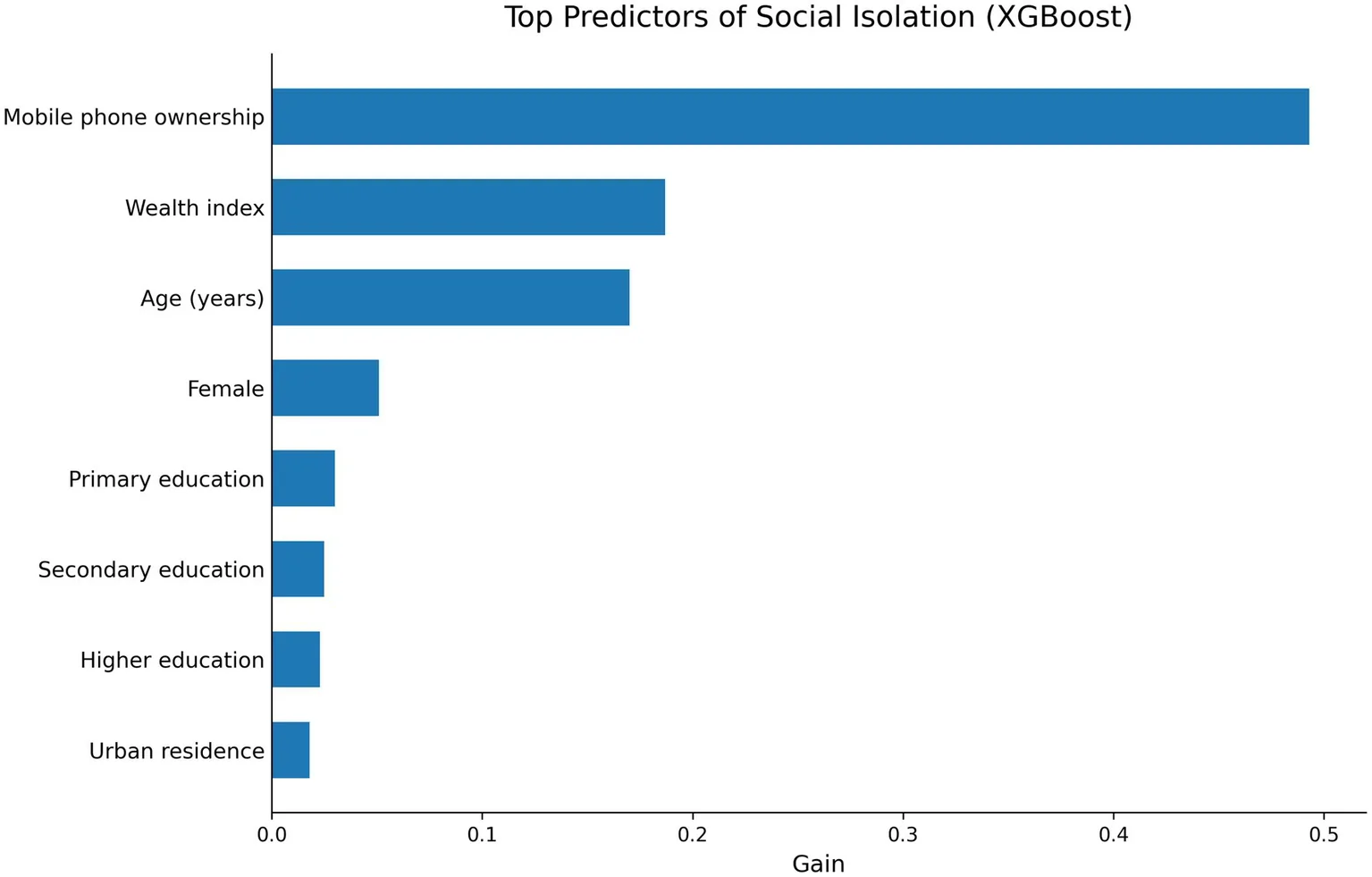
Relative importance of predictors in the XGBoost model for living alone among older adults aged ≥65 years, Rwanda DHS 2019–20.

### 3.7 Comparison of logistic regression and K-nearest neighbors results

The findings from the exploratory machine-learning analyses were generally consistent with those of the multivariable logistic regression model. In the logistic regression analysis, mobile phone ownership was significantly associated with lower odds of living alone after adjustment for potential confounders. Within the exploratory machine-learning analyses, mobile phone ownership consistently ranked highest according to the selected feature-importance metric.

The XGBoost model demonstrated slightly higher discrimination (AUC = 0.77) than the KNN model (AUC = 0.76), indicating a modest improvement in ranking performance. However, both models showed only moderate discriminative ability and limited classification performance. Therefore, the machine-learning analyses should be interpreted as complementary exploratory analyses that identify patterns and relative predictor importance rather than provide causal inference or clinically useful prediction.

Overall, the consistency between the regression and machine-learning analyses supports the robustness of the observed association between mobile phone ownership and living alone as a structural indicator of social isolation among older adults in Rwanda, while acknowledging the exploratory nature of the predictive models.

### 3.8 Summary of empirical findings

Across both the multivariable logistic regression and exploratory machine-learning analyses, mobile phone ownership was consistently associated with lower odds of living alone among older adults in Rwanda. Female sex and lower socioeconomic status were also associated with a greater likelihood of living alone, whereas education level and place of residence showed weaker or non-significant associations. Overall, the findings from the different analytical approaches provide converging evidence that mobile phone ownership was strongly associated with living alone as a structural indicator of social isolation in this dataset.

## 4 Discussion

This study provides nationally representative evidence on the association between digital access and living alone as a structural indicator of social isolation among older adults in Rwanda. Using data from the RDHS 2019–20 and combining regression-based inference with machine-learning approaches, the findings consistently indicate that mobile phone ownership is strongly associated with reduced likelihood of living alone in later life. This study contributes to a limited but growing body of evidence on digital access and structural dimensions of social isolation among older adults in sub-Saharan Africa. While most existing research has been conducted in high-income settings, our findings suggest that digital connectivity may play an important role in shaping structural dimensions of social integration in low-resource contexts.

This study provides evidence on the relationship between mobile phone ownership and structural dimensions of social isolation among older adults in Rwanda. The findings demonstrate a clear and substantial digital divide: Older adults without a mobile phone were substantially more likely to live alone (19.4%) than those living in households with a mobile phone (1.8%). This large bivariate difference highlights the potential relationship between digital access and living alone as a structural indicator of social isolation among older adults.

In unadjusted analyses, older adults who owned a mobile phone had substantially lower odds of living alone, highlighting a pronounced digital divide in household-based social integration. Importantly, this association persisted after adjustment for age, sex, wealth, education, and place of residence.

The multivariate logistic regression results reinforce this association. After adjusting for age, sex, wealth index, education, and residence, mobile phone ownership remained a strongly associated factor linked to lower likelihood of living alone. Older adults who owned a mobile phone had 87% lower odds of living alone (AOR = 0.13, 95% CI: 0.08–0.22). These findings suggest that mobile phone ownership is associated with living arrangements that reflect structural dimensions of social isolation beyond observed socio-demographic differences. These findings align with global evidence showing that mobile communication technologies help older adults maintain social ties, engage in community life, and access information and support networks (22, 23).

The strong inverse association between household mobile phone ownership and living alone should nevertheless be interpreted cautiously. Because the RDHS data are cross-sectional, the observed association does not establish whether household mobile phone ownership is related to subsequent differences in living arrangements or whether household living arrangements influence access to mobile phones. Selection effects may also contribute to the observed association, as households with different socioeconomic circumstances, family structures, or levels of social support may differ in both mobile phone ownership and living arrangements. Although the analysis adjusted for age, sex, wealth index, education, and place of residence, residual confounding cannot be excluded.

At the same time, the observed association may also reflect broader social and economic processes beyond digital access alone. Older adults living with others may benefit from greater family support, shared household resources, or assistance in acquiring and using mobile phones. Conversely, individuals living alone may be more likely to experience widowhood, financial constraints, reduced mobility, or limited social support, all of which could influence both living arrangements and access to communication technologies. Although the analysis adjusted for several socioeconomic factors, residual confounding and reverse association remain possible and should be considered when interpreting the findings.

The results also shed light on important demographic disparities. Women had significantly higher odds of living alone than men (AOR = 1.86), reflecting well-documented patterns of female longevity, widowhood, and gendered care giving dynamics in sub-Saharan Africa (24). This indicates that gender-sensitive approaches are essential when designing social and digital interventions for aging populations.

These findings are notable because the study focuses on living alone as a structural indicator and proxy measure of social isolation. Prior research suggests that digital technologies may exert stronger effects on objective aspects of social integration, such as frequency of contact or co-residence, than on subjective loneliness, which is shaped by psychological and relational factors (25). In contexts where extended family systems are changing due to migration and urbanization, mobile phones may help older adults remain embedded within family networks even when physical proximity is reduced.

Socioeconomic disparities observed in the adjusted model further underscore the intersection between social isolation and broader social inequalities. Higher wealth was associated with lower odds of living alone, suggesting that economic resources may facilitate shared living arrangements, stronger family support, or greater access to communication tools. These patterns echo findings from other African and global settings, where poverty and gendered life-course trajectories shape social connection in older age (26).

Other covariates, including age, education level, and urban/rural residence, were not statistically significant in the adjusted model. This suggests that, within the older population (65 years and older), living alone was not significantly associated with age group, education level, or place of residence after adjustment for wealth index and mobile phone ownership. The absence of a significant urban–rural difference indicates that geographic residence was not independently associated with living alone in this dataset, suggesting that digital connectivity may be a more decisive factor than geography alone, an encouraging sign given Rwanda’s extensive national broadband and 4G/LTE roll out.

This study provides nationally representative evidence of an association between mobile phone ownership and living alone as a structural indicator of social isolation among older adults in Rwanda. After adjustment for age, sex, wealth index, education level, and place of residence, mobile phone ownership remained independently associated with lower odds of living alone. These findings suggest that mobile phone ownership may be associated with household living arrangements that reflect structural dimensions of social isolation in later life.

The magnitude of the association observed is substantial. Older adults who owned a mobile phone had markedly lower odds of living alone, highlighting the potential of mobile connectivity to support structural aspects of social integration, such as maintaining family contact and facilitating shared living arrangements. This aligns with global evidence showing that digital connectivity strengthens social ties and reduces isolation among aging populations.

The higher odds of living alone observed among women likely reflect well-documented gendered patterns of widowhood, longer life expectancy, and caregiving dynamics in later life. Similarly, the protective effect of higher household wealth underscores the importance of economic resources in sustaining social support networks and access to communication tools. Together, these findings point to digital access as an emerging social determinant of health in later life, particularly in rapidly digitizing low- and middle-income settings.

The machine-learning analyses provided complementary exploratory insights alongside the primary regression-based findings. Both the K-nearest neighbors and XGBoost models demonstrated moderate discrimination (AUC ≈ 0.76–0.77), although classification performance at the selected thresholds was limited. Within these exploratory models, mobile phone ownership ranked highest according to the selected feature-importance metrics and was consistently identified as an important predictor of living arrangement status, followed by wealth index and age. These exploratory findings were broadly consistent with the regression analysis but should be interpreted as predictive rather than confirmatory evidence.

From a policy perspective, these findings highlight mobile phone ownership as a potentially important marker associated with living arrangements among older adults in Rwanda. The observed associations support further investigation into whether digital inclusion strategies could contribute to healthy ageing and social participation. Future longitudinal and interventional studies are needed to determine whether expanding digital access can influence living arrangements or broader dimensions of social connectedness. Given the observed socioeconomic gradient, future interventions may consider prioritizing economically disadvantaged older adults while evaluating their effectiveness prospectively.

Overall, the findings suggest that mobile phone ownership is associated with living arrangements among older adults and may represent an important indicator of digital inclusion in this population. These results provide a basis for future longitudinal and intervention studies to determine whether improving digital access can influence healthy ageing and broader dimensions of social connectedness.

### 4.1 Comparison with prior studies

Our findings align closely with international research demonstrating the role of digital tools in improving structural social connectedness. Studies in high-income countries have consistently shown that mobile phone access increases opportunities for communication, enhances perceived social support, and reduces loneliness among older adults (27). Similarly, work in China and South Korea indicates that mobile communication facilitates intergenerational connectivity and improves psychological well-being among older individuals (28).

Although empirical evidence from sub-Saharan Africa remains comparatively limited, available studies point in a similar direction. In South Africa, mobile phone ownership has been associated with stronger family connectedness and reduced loneliness among older adults (29). Research from Kenya and Uganda suggests that mobile phones support participation in religious, community, and economic activities, thereby contributing indirectly to social integration in older age (30). Likewise, a study in Ghana found that mobile phones serve as a primary means of communication with relatives, particularly for older adults who no longer co-reside with family members (31).

Similar to these findings, our results support the hypothesis that digital connectivity is an emerging social determinant of health in low- and middle-income settings, particularly where traditional family structures are shifting due to migration and urbanization.

Our study also contributes uniquely by providing population-level evidence in Rwanda, where digital access is rapidly expanding but aging-related research remains sparse. The magnitude of the protective effect found in this study (AOR = 0.13) is stronger than reported in several other LMIC studies, potentially reflecting Rwanda’s high penetration of mobile services and strong digital infrastructure.

### 4.2 Interpretation of covariates in context of existing literature

#### 4.2.1 Sex differences

The higher odds of living alone observed among women are consistent with patterns reported across sub-Saharan Africa. Women tend to outlive men, experience higher rates of widowhood, and may face greater economic and social vulnerability in older age, all of which increase the likelihood of living alone (24). These findings highlight the importance of considering gendered life-course dynamics when addressing social isolation among older populations.

#### 4.2.2 Wealth effects

The association between higher wealth and lower odds of living alone aligns with global evidence indicating that economic resources facilitate stronger social networks, more stable living arrangements, and greater access to communication tools. Studies conducted in Tanzania, Ethiopia, and Nigeria similarly report that lower socioeconomic status is associated with increased risk of isolation and reduced social support in older age (32, 33). In Rwanda, wealth-related disparities may influence the ability of older adults to maintain shared households or access digital technologies that support social connection.

#### 4.2.3 Non-significance of age, education, and residence

In contrast to some international studies where age or educational attainment predicts social isolation, these variables were not statistically significant in the adjusted model. This finding suggests that, within Rwanda’s older adult population, digital access and socioeconomic status may be more salient determinants of structural isolation than demographic characteristics alone. Strong cultural norms of communal living in both rural and urban settings may further attenuate differences by age or residence, underscoring the context-specific nature of social isolation.

### 4.3 Policy implications

The findings of this study have important implications for Rwanda’s aging and digital transformation agendas. National strategies such as the Smart Rwanda Master Plan and the National Digital Health Strategy emphasize equitable access to technology as a pathway to improved social and health outcomes. Integrating older adults into these initiatives may yield meaningful benefits for social connectedness and well-being.

Policy-relevant interventions may include expanding access to affordable mobile phones for older adults, developing age-friendly digital literacy programs, establishing community-level phone access points, and integrating digital engagement into existing social protection and community health worker (CHW) programs. Given the observed socioeconomic and gender disparities, targeted approaches focusing on poorer and female older adults may warrant further evaluation as part of broader strategies addressing social isolation and social connectedness.

### 4.4 Strengths and limitations

This study has several notable strengths. First, it draws upon nationally representative data from the Rwanda Demographic and Health Survey (DHS) 2019–20, ensuring that the findings generalize to older adults across the country. The large sample size of individuals aged 65 years and older (N = 2,081) provides robust statistical power and enhances the reliability of the estimates. Additionally, the analysis incorporates DHS sampling weights, which appropriately account for the complex multistage cluster sampling design and ensure unbiased population-level inferences. A further strength lies in the clear and consistent operationalization of living alone as a structural indicator of social isolation using household composition data, a commonly applied approach in demographic research where direct psychosocial indicators are unavailable.

However, several limitations should also be acknowledged. The primary outcome was living alone, which was used as a structural proxy for social isolation. This measure does not capture subjective dimensions such as emotional loneliness, perceived social support, or frequency of social interactions. These aspects may differ substantially even among individuals who live with others. The cross-sectional nature of the RDHS data also limits causal interpretation; it cannot be determined whether household mobile phone ownership is associated with living arrangements because access to a phone influences social connectedness, or whether older adults living with others are more likely to reside in households that own mobile phones. In addition, the mobile phone ownership variable (hv243a) captures household ownership of at least one mobile phone rather than personal ownership by the older adult. Consequently, older adults living in larger households may be classified as having household phone access even if they do not personally own or regularly use a mobile phone, which may have introduced some exposure misclassification.

Furthermore, mobile phone ownership does not reflect how frequently or effectively older adults use their devices, nor does it measure digital literacy, affordability of airtime or data, or accessibility of charging infrastructure. The machine-learning models demonstrated moderate discrimination but limited sensitivity, reflecting the challenges of predicting a relatively rare outcome and the complex, multidimensional nature of social connectedness and isolation. These findings suggest that while machine learning can support risk stratification, it may be less effective for identifying individuals who lived alone in population-based datasets. Also, machine-learning analyses used a single stratified 70/30 training–testing split rather than repeated cross-validation or bootstrap-based internal validation. Consequently, the reported predictive performance may be sensitive to the particular split and should be interpreted as exploratory rather than as definitive estimates of model performance. Finally, the analysis did not include several potentially relevant confounders, such as disability status, chronic illness, mobility limitations, or cognitive impairment, which may influence both living arrangements and technology access. Additionally, occupational variables available in the DHS dataset were reviewed; however, they were not included in the final analysis due to substantial heterogeneity and missingness among older adults aged 65 years and above.

Despite these limitations, the study has several important strengths, including the use of nationally representative RDHS data, application of sampling weights to account for the complex survey design, and the explicit operationalization of living alone as a structural indicator of social isolation. Nevertheless, the findings should be interpreted in light of the cross-sectional design, the use of household mobile phone ownership as the exposure measure, and the reliance on a structural proxy rather than comprehensive measures of social isolation or loneliness. Future longitudinal studies incorporating individual-level measures of digital access and multidimensional assessments of social connectedness are needed to further investigate these associations.

## 5 Conclusion

This study provides nationally representative evidence that household mobile phone ownership was associated with living alone as a structural indicator of social isolation among older adults in Rwanda. Using data from RDHS 2019–20 and integrating multivariable logistic regression with complementary exploratory machine-learning analyses, the study found that older adults living in households with a mobile phone had lower odds of living alone after adjustment for sociodemographic and socioeconomic characteristics.

The regression and exploratory machine-learning analyses provided converging evidence that household mobile phone ownership was consistently associated with living arrangement status in this dataset. In the exploratory XGBoost model, household mobile phone ownership ranked highest according to the selected feature-importance metric, while wealth index and age also contributed to prediction. These findings should be interpreted in light of the cross-sectional study design and the use of household mobile phone ownership and living alone as proxy measures.

The findings highlight the importance of considering digital inclusion alongside broader social and economic factors in research on healthy ageing. However, digital access should not be viewed as a standalone solution to structural social isolation. Rather, it should be considered alongside complementary measures addressing poverty, income security, housing, health care access, rehabilitation services, and broader social protection for older adults.

Future longitudinal and intervention studies are needed to determine whether improving digital access influences living arrangements, social connectedness, or healthy ageing outcomes among older adults. Such evidence would help clarify the potential role of digital inclusion within comprehensive healthy ageing and social protection strategies in Rwanda and other low- and middle-income countries.

## Data Availability

Publicly available datasets were analyzed in this study. The data used in this study are available from the Demographic and Health Surveys (DHS) Program at https://www.dhsprogram.com/data/. Access to the Rwanda Demographic and Health Survey (RDHS) 2019–20 dataset can be obtained upon reasonable request through the DHS Program website. No accession number is required.
